# LGR5 is required for the maintenance of spheroid-derived colon cancer stem cells

**DOI:** 10.3892/ijmm.2014.1752

**Published:** 2014-04-24

**Authors:** XIANG CHEN, BO WEI, XIAOYAN HAN, ZONGHENG ZHENG, JIANGLONG HUANG, JIANPEI LIU, YONG HUANG, HONGBO WEI

**Affiliations:** 1 Department of Gastrointestinal Surgery, The Third Affiliated Hospital of Sun Yat-sen University, Guangzhou, Guangdong 510630, P.R. China; 2 Central Laboratory, The Third Affiliated Hospital of Sun Yat-sen University, Guangzhou, Guangdong 510630, P.R. China

**Keywords:** LGR5, maintenance, spheroid cells, colon cancer stem cells

## Abstract

Colon cancer stem cells (CCSCs) are involved in colon cancer and promote tumor progression and recurrence. LGR5, a marker for intestinal stem cells (ISCs), is also considered to serve as a marker for CCSCs. However, the precise function of LGR5 in CCSCs is unclear. In this study, we demonstrated that LGR5 was highly expressed in CCSCs-enriched HT29 spheroid cells. Downregulation of LGR5 with small interfering RNA (siRNA) decreased the expression of stem the cell markers CD133 and CD44 in HT29 spheroid cells. In addition, silencing of LGR5 inhibited cell proliferation, secondary tumor sphere formation and induced cell apoptosis, and G0/G1 phase arrest *in vitro* by modulating Bcl-2, Bcl-xL and Bax. Knockdown of LGR5 enhanced chemosensitivity and reduced the invasive ability of HT29 spheroid cells. Moreover, LGR5-siRNA suppressed tumorigenicity of HT29 spheroid cells *in vivo*. The findings suggested that LGR5 plays a vital role in the maintenance of CCSCs and is a potential therapeutic target for colon cancer.

## Introduction

Colon cancer is a common malignancy worldwide with a high incidence of tumor recurrence and metastasis, resulting in cancer-related mortalities (1). Despite advances in therapy, including surgery and chemotherapy, tumor recurrence and metastasis cannot be effectively prevented (2).

Accumulating evidence suggests that a small subpopulation of cancer cells known as cancer stem cells (CSCs), which exist in various types of cancer and are characterized by extensive ability of self-renewal and differentiation, and have a high potential for tumor propagation and therapy-resistance, may contribute to tumor progression, recurrence and metastasis (3,4).

Targeting therapy towards colon CSCs may be a promising approach to eradicating colon cancer more efficiently. Several molecular markers have been previously employed to identify CCSCs, such as CD133, CD44, ALDH and EpCAM (5–8). However, the specificity of these markers are questionable (9). LGR5, a Wnt target gene, was initially identified as a marker of intestinal stem cells (ISCs). LGR5^+^ cells are located at the crypt base and generate various types of differentiated epithelial cells in the intestine to maintain the self-renewal and homeostasis of intestinal mucosa (10–12). CCSCs are considered to originate from normal ISCs (13–15). Conditional deletion of APC exclusively in the LGR5^+^ ISCs of a murine model led to the rapid growth and spread of large adenomas in the small intestine and colon, whereas deletion of Apc in the non-stem cell compartment, resulted in the growth inhibition of adenomas (15). Therefore, LGR5 may also serve as a marker for CCSCs (16). A higher expression of LGR5 has been found in colon cancer and adenomas relative to matched normal mucosa, and is associated with malignant clinicopathological characteristics, suggesting that LGR5 is involved in tumor development and progression (17,18).

Although LGR5 is regarded as a potential marker for CCSCs, little is known concerning its function. We previously reported that colon cancer spheroid cells derived from serum-free culture possessed stem-like properties, including higher proliferative, migratory, invasive, and metastatic ability, and were thus considered CCSCs-enriched models (19). In this study, we detected the expression of LGR5 in these spheroid cells, and assessed its role in CCSCs.

## Materials and methods

### Cell culture

The HT29 human colon cancer cell line (ATCC, HTB-38) was maintained in DMEM/F12 with 10% fetal bovine serum (FBS). For the sphere culture, HT29 cells were grown at a density of 2×10^6^ cells/ml in 100 mm ultra-low attachment dishes (Corning Life Sciences, Oneonta, NY, USA) in serum-free DMEM/F12 medium (SFM) containing 2% B27 (Invitrogen, Carlsbad, CA, USA), 20 ng/ml epidermal growth factor (EGF), 10 ng/ml basic fibroblast growth factor (bFGF) (both from Peprotech Inc., Rocky Hill, NJ, USA), 5 μg/ml routine insulin (Invitrogen). The cells were incubated in a humidified atmosphere at 37°C with 5% CO_2_. To induced differentiation *in vitro*, spheroid cells were cultured in DMEM/F12 supplemented with 10% FBS for 48 h and then harvested for assays.

### Immunofluorescent staining

Spheroid cells were cytospun onto glass slides, fixed with 4% paraformaldehyde for 10 min, and permeablized with 0.1% Triton X-100 for 15 min. Adherent and differentiated cells were cultured on sterile cover slips in 6-well plates for 48 h and fixed as described above. The cells were incubated with the primary anti-LGR5 Ab antibody (Abcam, Cambridge, UK) at 4°C overnight, followed by incubation with secondary DyLight-conjugated anti-rabbit Ab antibody (Abcam) for 1 h at room temperature. DAPI (Invitrogen) was used to counterstain the nuclei. Fluorescent images were captured using a Zeiss confocal microscope (LSM-710; Zeiss, Jena, Germany).

### Small interfering RNA (siRNA) transfection

siRNA were obtained from GenePharma Co., Ltd. (Suzhou, China). Primer sequences of LGR5-siRNA were: forward, 5′-GCUCCA GCAUCACUUAUGATT-3′; and reverse, 5′-UCAUAAGUG AUGCUGGAGCTT-3′. Dissociated HT29 spheroid cells (5×10^5^) were seeded in 6-well plates in SFM. Twenty-four hours later, siRNA were transfected into spheroid cells at a final concentration of 100 nM using Lipofectamine RNAiMAX reagent (Invitrogen) according to the manufacturer’s instructions. Spheroid cells were either transfected or not transfected with scrambled siRNA and used as the blank or negative control (NC). The cells were collected for a series of experiments at 48 h after transfection.

### Quantitative RT-PCR (qRT-PCR)

Total RNA was extracted using RNAiso Plus (Takara Bio, Inc., Shiga, Japan) according to the manufacturer’s instructions. cDNAs were synthesized using PrimeScript RT reagent kit (Takara) with 1 μg total RNA for each sample. The RT-PCR reaction was performed using ABI 7500 Fast (Applied Biosystems, Foster City, CA, USA) with SYBR-Green I reagents (Takara). The cycle condition were: denaturation at 95°C for 30 sec, 40 amplification cycles at 95°C for 3 sec and 60°C for 30 sec. β-actin was used as the control. Primer sequences used were: LGR5 forward, 5′-GAGGATCTGGTGAGCCTGAGAA-3′; and reverse, 5′-CATAAGTGATGCTGGAGCTGGTAA-3′; β-actin forward, 5′-CAACTGGGACGACATGGAGAAA-3′; and reverse, 5′-GATAGCAACGTACATGGCTGGG-3′. The results were analyzed by the 2^−ΔΔct^ method.

### Western blotting

Cells were lysed in RIPA buffer with 10% phenylmethylsulfonyl fluoride. The cell extracts were loaded on 10% SDS-polyacrylamide gels and transferred onto polyvinylidene fluoride membranes. The membranes were blocked for 1 h at room temperature with 5% non-fat milk in TBST, and then incubated with anti-LGR5 (diluted at 1:100; Abgent, San Diego, CA, USA), anti-Bcl-2 (diluted at 1:1,000), anti-Bcl-xL (diluted at 1:1,000), anti-Bax (diluted at 1:1,000) (all from Cell Signaling Technology, Inc., Danvers, MA, USA) at 4°C overnight. Following incubation with HRP-conjugated secondary antibody (diluted at 1:1,000; Santa Cruz Biotechnology, Inc., Santa Cruz, CA, USA ), immuno-complexes were visualized by an enhanced chemiluminescence detection system (Millipore Corp., Billerica, MA, USA). Endogenous GADPH was used for normalization.

### Detection of surface markers LGR5, CD133 and CD44 by flow cytometry

Cells were dissociated and washed twice in PBS. Subsequently, cell suspensions were incubated with 1:50 PE-conjugated mouse anti-LGR5 Ab (OriGene, Rockville, MD, USA), or 1:10 PE-conjugated mouse anti-CD133 (Miltenyi Biotec, Bergisch Gladbach, Germany) and 1:10 FITC-conjugated mouse anti-CD44 (BD Biosciences, Franklin Lakes, NJ, USA) antibodies for 20 min in the dark. The cells were then washed twice in cold PBS with 1% BSA and resuspended in 300 μl cold PBS with 1% BSA for flow cytometric analysis within 1 h.

### Proliferation and chemo-sensitivity assays

Cell proliferation and chemosensitivity assays were determined using Cell Counting kit-8 (CCK-8; Dojindo Laboratories, Kumamoto, Japan). Spheroid cells were dissociated and seeded in 96-well plates at a density of 2×10^3^ cells/well in 100 μl SFM overnight, and then transfected with 100 nM of indicated siRNA. At 0, 1, 2, 3 and 4 days after transfection, 10 μl CCK-8 reagent was added to each well and the culture was incubated for a further 4 h. The optical density (OD) value in each well was measured by a microplate reader at a wavelength of 450 nm. For chemosensitivity assay, cells (4×10^3^) were seeded in each well and treated with various concentrations of 5-Fu (0, 50, 100 and 200 μg/ml) or cisplatin (0, 12, 24 and 48 μg/ml) (Sigma, St. Louis, MO, USA) 48 h post-transfection. After incubation for 48 h, a CCK-8 assay was performed and the survival rate of cells was calculated as: OD_treatment_/OD_control_ × 100%. Experiments were performed in triplicate.

### Sphere formation assay

After 48 h transfection, spheroid cells were dissociated and seeded in 24-well ultra-low attachment plates (Corning Life Sciences) at a density of 2×10^3^ cells/well in 500 μl SFM and then grown for a further 7 days. Spheres >50 μm were counted by microscope.

### Apoptosis assay

Apoptotic cell rates were measured by Annexin V and propidium iodide (PI) double staining using an Annexin V/FITC kit (KeyGen, Nanjing, China). Spheroid cells were collected and dissociated 48 h after transfection, washed with cold PBS containing 2% BSA, resuspended in binding buffer, and then incubated with Annexin V-FITC and PI for 30 min in the dark at room temperature. The staining cells were analyzed using FACSCanto II flow cytometer (BD Biosciences).

### Cell cycle assay

Spheroid cells were collected 48 h after transfection. Cells (1×10^6^) were fixed with cold 70% ethanol at 4°C overnight, washed with cold PBS, and stained with PI (50 μg/ml) in PBS containing RNase (50 μg/ml) (both from Sigma) in the dark for 30 min. Analyses were performed by FACSCanto II flow cytometer (BD Biosciences).

### Invasion assay

Invasion assay was performed using 24-well plate Transwell chambers with 8 μm-pore polycarbonate filter inserts (Corning Life Sciences). A total of 48 h post-transfection, spheroid cells were detached and 1×10^5^ cells in 100 μl DMEM/F12 without FBS were seeded onto the upper chamber coated with Matrigel (BD Biosciences). Then, 600 μl DMEM/F12 medium containing 10% FBS was added into the lower chamber. After incubation for 48 h, the cells on the upper side of the membrane were removed and the cells that migrated to the underside were fixed in 4% paraformaldehyde, stained with 0.1% crystal violet and counted in five random fields under microscopy.

### In vivo tumorigenesis

Spheroid cells were detached and resuspended in PBS. Cells (2×10^6^) in 100 μl PBS were injected subcutaneously into the flanks of 5-week-old male BALB/C-nu mice (Beijing HFK Bioscience, Beijing, China). When the tumor volume reached 80–100 mm^3^, the mice were randomly divided into the NC and LGR5-siRNA groups (n=4 mice per group). The NC and LGR5-siRNA groups were administered intratumoral injection of 50 μg scrambled siRNA and LGR5-siRNA, respectively, each week. The diameters of subcutaneous tumors were measured with a caliper every 4 days, and the tumor volume was calculated using the formula: volume = width^2^ × length × 0.5. Four weeks later, the mice were sacrificed and tumors were extracted and weighed. The experiments were performed in accordance with the Guide for the Care and Use of Laboratory Animals published by the National Institutes of Health.

### Statistical analysis

Results were presented as means ± SD. Statistical analysis was performed using one-way ANOVA or the Student’s t-test. The LSD method was used for multiple comparisons. P<0.05 was considered to be statistically significant.

## Results

### LGR5 is overexpressed in HT29 spheroid cells

**Figure 1:**
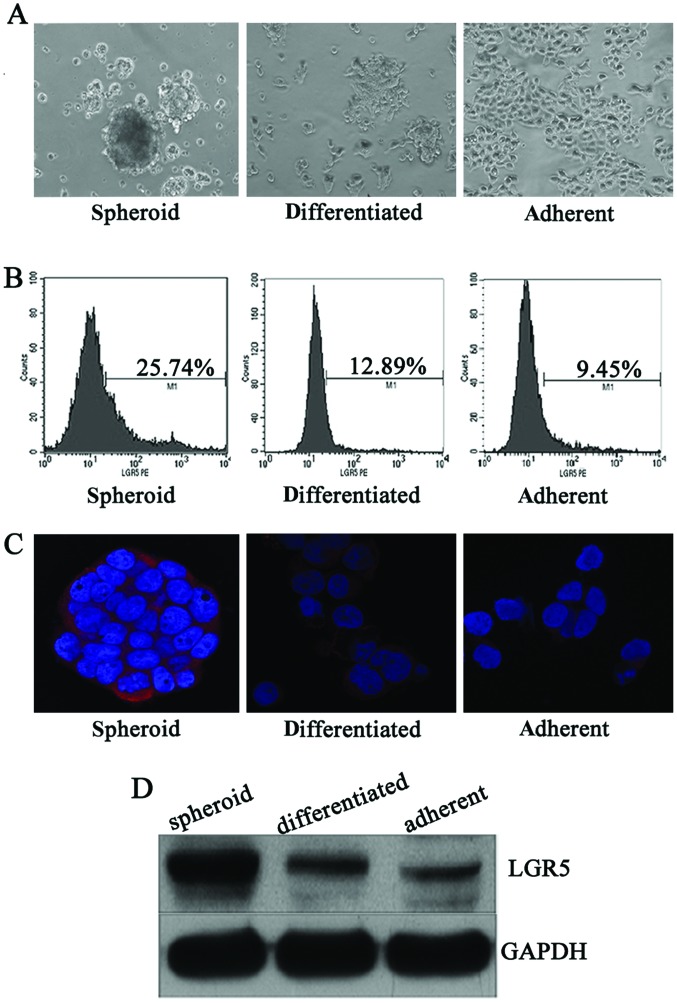
LGR5 expression in spheroid, differentiated and adherent HT29 cells. (A) Morphology of spheroid, differentiated and adherent HT29 cells. When the adherent HT29 cells grew in serum-free DMEM/F12 medium (SFM), they became suspended and turned into large spheres, but after being reseeded in medium containing FBS, the cells were differentiated into adherent cells again (x100). (B) Flow cytometric analysis of LGR5^+^ cell subpopulation in HT29 spheroid, differentiated and adherent cells. (C) Immunofluorescent staining of LGR5 in HT29 spheroid, differentiated and adherent cells. LGR5 staining is red and nuclei are stained in blue. (D) LGR5 protein level in HT29 spheroid, differentiated and adherent cells detected by western blotting.

LGR5, also known as GPR49, is a member of the G protein-coupled receptor (GPCR) family of proteins, and enhances Wnt signaling by binding with R-spondin (10,20). Therefore, to obtain comprehensive expression profile in CCSCs, we detected the extra- and intracellular expression of LGR5 in HT29 spheroid cells and their counterparts, which were differentiated *in vitro* and cultured in monolayer (Fig. 1A). Flow cytometric analysis revealed that HT29 spheroid cells contained a high proportion of LGR5^+^ cells, while differentiated and adherent cells had a smaller LGR5^+^ fraction (Fig. 1B). Similarly, compared with adherent counterparts, stronger cytoplastic staining and a higher LGR5 protein level was confirmed by immunofluorescent staining and western blotting in HT29 spheroid cells, respectively, which were significantly attenuated after inducing differentiation *in vitro* (Fig. 1C and D). These results suggest that LGR5 is associated with dedifferentiation of CCSCs.

### Expression of stem cell markers CD133 and CD44 is decreased following siRNA-mediated LGR5 knockdown in HT29 spheroid cells

**Figure 2:**
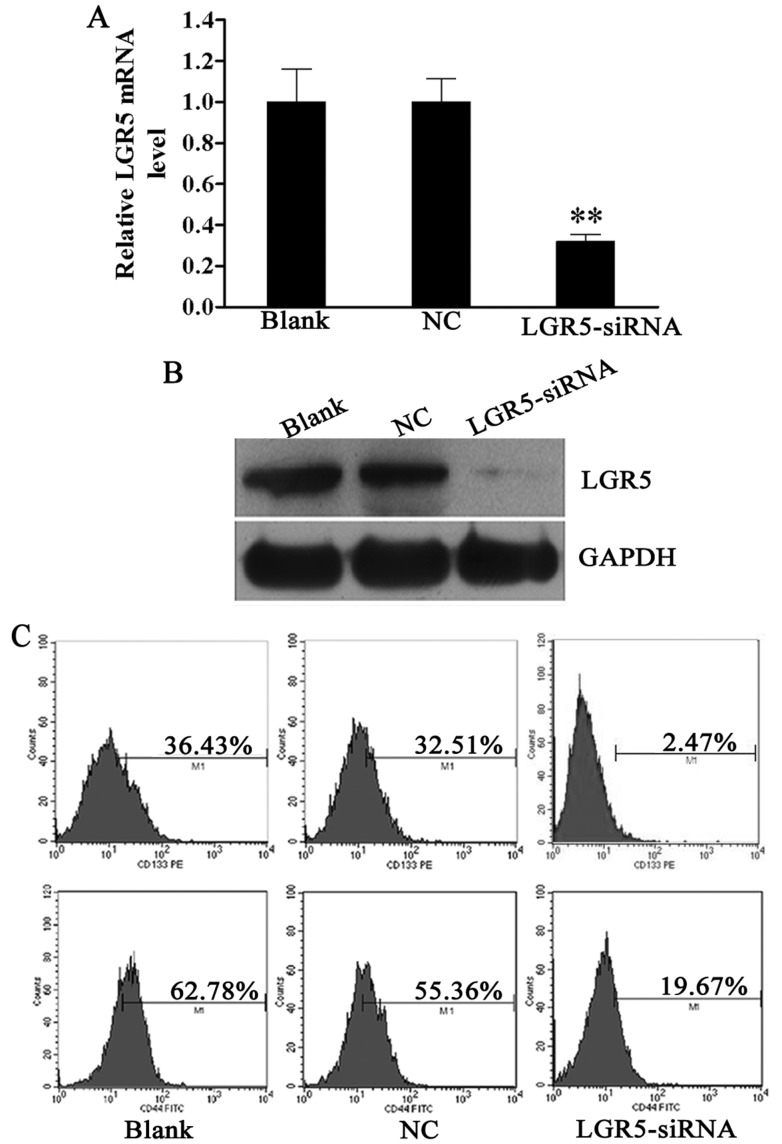
Expression of stem cell markers CD133 and CD44 is downregulated after LGR5 knockdown in HT29 spheroid cells. (A) Relative LGR5 mRNA level of HT29 spheroid cells at 48 h after small interfering RNA (siRNA) transfection was determined by quantitative RT-PCR (qRT-PCR). (B) Forty-eight hours post-transfection, LGR5 protein expression in HT29 spheroid cells was assessed by western blotting. (C) Expression of CD133 and CD44 was detected by flow cytometry. One of three similar experiments is shown. ^**^P<0.01 vs. negative control (NC) or blank group.

Since a high expression of LGR5 was detected in HT29 spheroid cells, we investigated the biological function of LGR5 in these cells. siRNA was used to knock down the expression of LGR5 in spheroid cells. LGR5 mRNA was downregulated by 68.2% in HT29 spheroid cells at 48 h after LGR5-siRNA transfection compared to the blank control (Fig. 2A). Moreover, western blotting confirmed that the LGR5 protein expression was also markedly reduced in HT29 spheroid cells transfected with LGR5-siRNA compared with the NC and blank controls (Fig. 2B).

In a previous study, we confirmed that HT29 spheroid cells were rich in CD133^+^ and CD44^+^ cells, which represented the subpopulation with stem-like properties (19). The effect of LGR5 on these cell populations was then examined. Forty-eight hours post-transfection, flow cytometric analysis revealed that the percentages of CD133^+^ and CD44^+^ cells were decreased in the LGR5-siRNA group (2.47 and 19.67%), as compared to the NC group (32.51 and 55.36%) and blank group (36.43 and 62.78%) (all P<0.01, Fig. 2C). These data reveal that LGR5 plays a key role in sustaining the stemness property of CCSCs.

### Downregulation of LGR5 expression impairs survival of HT29 spheroid cells in vitro

**Figure 3:**
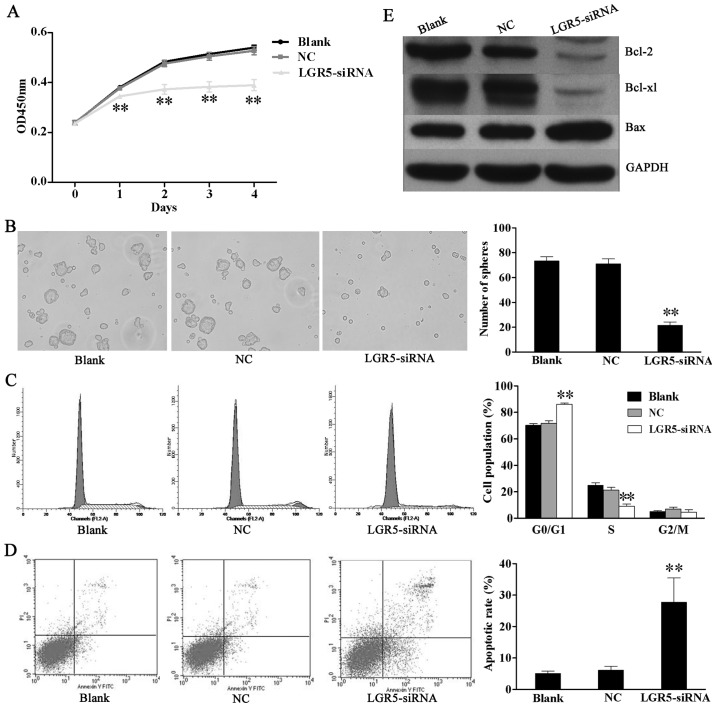
Effect of LGR5 knockdown on survival of HT29 spheroid cells. (A) Growth curves of the blank control, negative control (NC), LGR5-small interfering RNA (siRNA) transfected cells were determined by Cell Counting kit-8 (CCK-8) assay. (B) Tumor sphere formation assay after LGR5 silencing in HT29 spheroid cells (x100). (C) Cell cycle assay assessed by flow cytometry. (D) Cell apoptosis assay determined by flow cytometry of Annexin V/PI staining. (E) Expression of Bcl-2, Bcl-xL and Bax was evaluated by western blotting. Data are presented as means ± SD from three experiments. ^**^P<0.01 vs. the NC or blank group.

In order to investigate the effect of LGR5 on survival of CCSCs, proliferation, tumor sphere formation, cell cycle and apoptosis assays were performed in LGR5-siRNA transfected HT29 spheroid cells. CCK-8 assay showed that LGR5-siRNA cell growth was slower than the NC and blank control cells (Fig. 3A). In addition, LGR5 silencing suppressed the self-renewal of HT29 spheroid cells. LGR5-siRNA cells formed smaller and fewer secondary tumor spheres than the NC and blank control cells (Fig. 3B). The cell cycle assay revealed that the percentage of cells at the G0/G1 phase were significantly increased in the LGR5-siRNA group (86.23±0.85%) compared to the NC group (70.19±1.35%) and blank group (71.84 ± 1.78%), while those at S phase were markedly decreased in LGR5-siRNA group (9.16±1.62%) relative to the NC group (21.21±2.13%) and blank group (24.77±2.02%) (P<0.01, Fig. 3C). On the other hand, the LGR5-siRNA group had a higher apoptotic rate (27.7±7.74%) than the NC group (6.06±1.34%) and blank group (5.11±0.77%) (P<0.01, Fig. 3D). Furthermore, we analyzed the expression of survival-related genes including Bcl-2, Bcl-xL and Bax. Western blotting revealed that the expression of anti-apoptotic Bcl-2 and Bcl-xL genes was downregulated while the expression of the pro-apoptotic Bax gene was upregulated following LGR5 knockdown in HT29 spheroid cells (Fig. 3E). These results show that LGR5 may promote the spheroid cells survival by modulating the intrinsic apoptotic signaling pathway.

### Downregulation of LGR5 expression suppresses invasion and increases the chemosensitivity of HT29 spheroid cells

**Figure 4:**
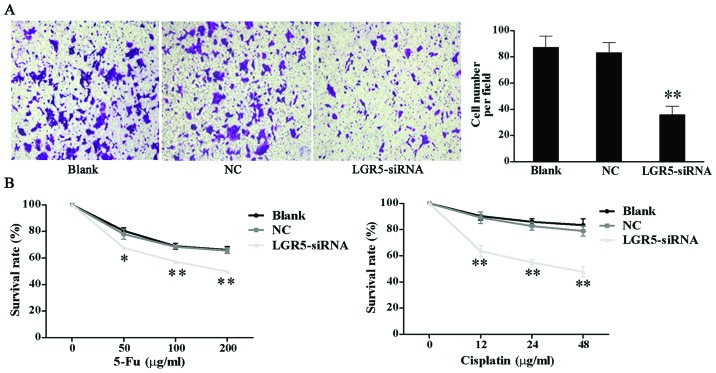
Downregulation of LGR5 suppressed invasive ability and increased chemosensitivity of HT29 spheroid cells. (A) Transwell invasion assay of blank, negative control (NC) and LGR5-small interfering RNA (siRNA) group. Cells that invaded through the Matrigel-coated filter were fixed, stained and counted (x100). (B) After treating with different concentrations of 5-Fu or cisplatin, survival rates of the blank, NC and LGR5-siRNA group were determined by Cell Counting kit-8 (CCK-8) assay. Data are presented as means ± SD from three experiments. ^*^P<0.05, ^**^P<0.01 vs. the NC or blank group.

As enhanced invasive ability and chemotherapy resistance are critical features of CSCs, we examined whether LGR5 affected these features in colon cancer. Results of matrigel invasion assays showed that the number of LGR5-siRNA cells (35.87±6.59) that invaded the underside of the membrane was significantly less than that of the NC (83.2±7.71) and blank control cells (87.27±8.57) (P<0.01, Fig. 4A).

To evaluate the effect of LGR5 on the drug resistance of spheroid cells, we conducted a chemosensitivity assay. Spheroid cells were treated with various concentration of cisplatin and 5-Fu 48 h post-transfection. After incubation for further 48 h, CCK-8 assay demonstrated that the survival rates of LGR5-siRNA transfected cells were significantly reduced compared with the NC and blank control cells at the respective concentrations (Fig. 4B).

### LGR5-siRNA attenuates tumorigenicity of HT29 spheroid cells in vivo

**Figure 5:**
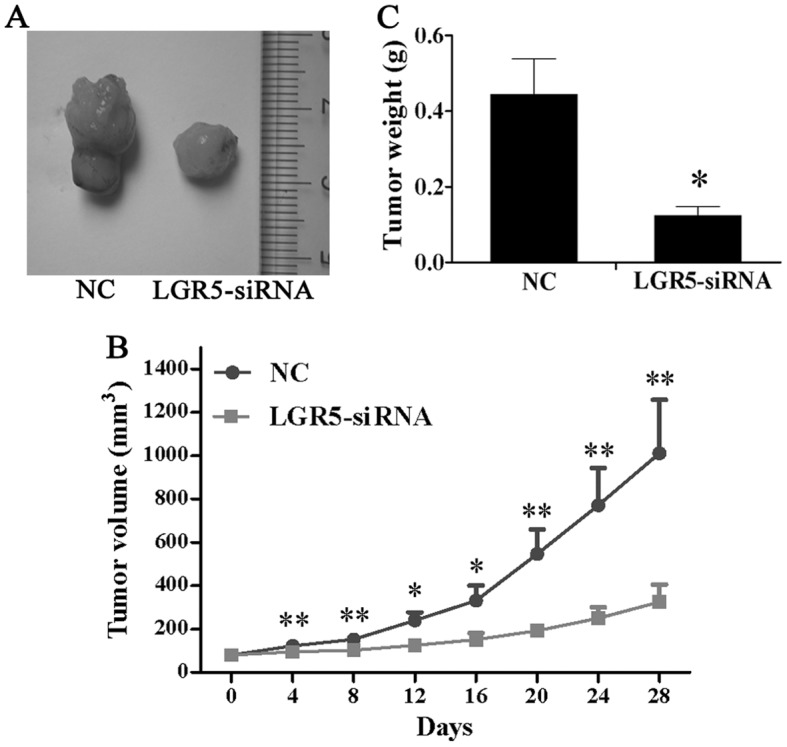
LGR5-small interfering RNA (siRNA) inhabited tumorigenic ability of HT29 spheroid cells *in vivo*. (A) The image of tumors extracted from mice in the LGR5-siRNA and negative control (NC) groupd. (B) The growth curves showed that LGR5-siRNA resulted in a marked decrease in tumor volume compared with the NC group. (C) Significant reduction in average tumor weight was observed in the LGR-siRNA group, as compared to the NC group. Data are presented as means ± SD. ^*^P<0.05, ^**^P<0.01 vs. NC group.

To assess the function of LGR5 for tumorigenicity *in vivo*, LGR5-siRNA were injected into the subcutaneous tumors in nude mice triggered by HT29 spheroid cells and tumor volume was measured every 4 days. As shown in Fig. 5A and B, mice in the LGR5-siRNA group developed much smaller subcutaneous tumors than those in the NC group. Four weeks after injection, the tumors were excised and weighed. The result showed that the average tumor weight of the LGR5-siRNA group was significantly lower than that of the NC group (Fig. 5C). These findings showed that LGR5-siRNA suppressed tumor growth *in vivo* in HT29 spheroid cells.

## Discussion

In colon cancer, a small fraction of tumor cells termed CSCs, which possess the ability of multi-differentiation and self-renewal, are suggested to be responsible for tumor initiation and growth. Therapies targeting CCSCs would likely result in more complete tumor degeneration. Identification of CCSCs is therefore a critical step for these targeting therapies. LGR5, known as an intestinal stem cell marker, has been considered a marker for CCSCs (15,16). Previous studies have shown that LGR5 was overexpressed in colon cancer tissue (17,18). However, the expression of LGR5 in CCSCs is unclear. In this study, we identified an increased expression of LGR5 on the surface of spheroid cells by flow cytometry, indicating that these CSCs-enriched colon cancer cells contained a high proportion of LGR5^+^ cells. Compared with CD133 expression in spheroid cells we reported previously (19), LGR5 expression may serve as a marker for a smaller heterogeneous population in spheroid cells. More importantly, recent studies have demonstrated that almost all of the LGR5^+^ cells isolated from xenografts, generated by the sorted LGR5^+^ colon cancer cells, were positive for CD133 and CD166. In addition, LGR5^+^/CD133^+^ cells formed more colonies than LGR5^−^/CD133^+^ cells (21). Findings of recent studies showed that the overlapping expression of putative stem cell markers CD133, CD44 and LGR5 in particular areas of gastric and intestinal mucosa, and concluded that they may be functionally associated (22,23). Our findings that LGR5 knockdown resulted in a decreased expression of CD133 and CD44 in spheroid cells confirm this conclusion, although the underlying molecular mechanism involved remains to be determined. These observations suggest that LGR5 may serve as a robust marker for CCSCs.

LGR5 belongs to the GPCR family and is a target gene of Wnt signaling that regulates tumorigenesis in colon cancer (24). Transformation exclusively occurring in LGR5^+^ cells could induce growth of colon adenomas (15). These findings show that LGR5 may be important in CCSCs. This study aimed to assess the function of LGR5 in CCSCs. As such, we examined the intracellular expression of LGR5 in CCSCs-enriched HT29 spheroid cells. The results showed that a high level of LGR5 was detected in the cytoplasm of HT29 spheroid cells, but was markedly decreased when the spheroid cells were induced to differentiate by culturing in FBS-containing medium. These preferential expression patterns suggest that LGR5 was correlated with the maintenance of HT29 spheroid cells. We downregulated LGR5 expression in spheroid cells by siRNA and examined whether it would affect the cells. First, we observed that LGR5 knockdown significantly inhibited the proliferation and secondary tumor sphere formation of HT29 spheroid cells *in vitro*. In addition, tumor growth *in vivo* was also suppressed by LGR5 siRNA. In order to clarify the mechanism of these effects, apoptosis and cell cycle assays were performed. The results showed that LGR5 silencing induced apoptosis and G0/G1 phase arrest in spheroid cells. These data show that LGR5 is essential for survival and the self-renewal of CCSCs, which is consistent with the findings in brain CSCs (25).

High potential for invasion and drug resistance are two important properties of CSCs (3,26). Recent studies have demonstrated that a high LGR5 expression in colon cancer tissues was closely associated with increased lymph node invasion, distant metastasis and poor chemotherapy response (18,27). These findings suggest that LGR5 exerts an effect on the malignant profile of CCSCs. To verify this hypothesis, invasion and chemosensitivity assays were conducted. As expected, LGR5 knockdown significantly reduced invasive ability and increased chemosensitivity in spheroid cells.

The molecular mechanism of LGR5 in modulating CCSCs properties remains to be determined. Results of a recent study showed that Rac1-ROS-NF-κB axis is critical for malignant transformation of LGR5^+^ ISC (28). Focal adhesion kinase (FAK), nuclear factor-κB (NF-κB), and c-fos are regulated by LGR5 through the Rho signaling pathway (29). These downstream targets, which are key factors for inflammation and cell adhesion, also play an important role in self-renewal and differentiation and migration of stem cells (30,31). Therefore, the crosstalk between LGR5 and other signaling pathways may contribute to maintaining the function of CCSCs. In this study, we observed that LGR5 silencing altered the expression of Bcl-2, Bcl-xL and Bax in HT29 spheroid cells, suggesting LGR5 is a potential regulator of apoptosis-related genes, although the underlying mechanism involved remains to be determined.

In conclusion, the present study has shown that LGR5 is overexpressed in spheroid-derived CCSCs. LGR5 knockdown suppressed growth, self-renewal, invasion and drug resistance, and induced apoptosis and G0/G1 phase arrest of spheroid cells *in vitro*, as well as the expression of stem cell markers CD133 and CD44, attenuating their tumorigenicity *in vivo*. LGR5 is therefore indispensable for the maintenance of CCSCs. Targeting LGR5 may be an effective therapeutic strategy for eliminating colon cancer.
